# Effect of Prenatal Exposure to Airborne Polycyclic Aromatic Hydrocarbons on Neurodevelopment in the First 3 Years of Life among Inner-City Children

**DOI:** 10.1289/ehp.9084

**Published:** 2006-04-24

**Authors:** Frederica P. Perera, Virginia Rauh, Robin M. Whyatt, Wei-Yann Tsai, Deliang Tang, Diurka Diaz, Lori Hoepner, Dana Barr, Yi-Hsuan Tu, David Camann, Patrick Kinney

**Affiliations:** 1 Columbia Center for Children’s Environmental Health, Mailman School of Public Health, Columbia University, New York, New York, USA; 2 Department of Statistics, National Cheng Kung University, Taiwan, Republic of China; 3 Centers for Disease Control and Prevention, National Center for Environmental Health, Division of Laboratory Sciences, Atlanta, Georgia, USA; 4 Department of Analytical and Environmental Chemistry, Southwest Research Institute, San Antonio, Texas, USA

**Keywords:** air pollution, neurodevelopment, polycyclic aromatic hydrocarbons, prenatal

## Abstract

Our prospective cohort study of nonsmoking African-American and Dominican mothers and children in New York City is evaluating the role of prenatal exposure to urban pollutants, including polycyclic aromatic hydrocarbons (PAHs), environmental tobacco smoke (ETS), and pesticides, in the pathogenesis of neurobehavioral disorders. We used the Bayley Scales of Infant Development to evaluate the effects on child mental and psychomotor development of prenatal exposure to airborne PAHs monitored during pregnancy by personal air sampling. Behavioral development was assessed by the Child Behavior Checklist. We adjusted for potential confounders including sociodemographic factors and prenatal exposure to ETS and chlorpyrifos. Prenatal exposure to PAHs was not associated with psychomotor development index or behavioral problems. However, high prenatal exposure to PAHs (upper quartile) was associated with lower mental development index at age 3 [β= –5.69; 95% confidence interval (CI), –9.05 to –2.33; *p* < 0.01]. The odds of cognitive developmental delay were also significantly greater for children with high prenatal exposure (odds ratio = 2.89; 95% CI, 1.33 to 6.25; *p* = 0.01). General estimated equation analysis showed a significant age × PAH effect on mental development (*p* = 0.01), confirming the age-specific regression findings. Further adjustment for lead did not alter the relationships. There were no differences in effect sizes by ethnicity. The results require confirmation but suggest that environmental PAHs at levels recently encountered in New York City air may adversely affect children’s cognitive development at 3 years of age, with implications for school performance.

The impact of environmental toxicants on children’s health is increasingly recognized as significant (Faustman 2000; Greater Boston Physicians for Social Responsibility 2000; Landrigan et al. 1999; Perera et al. 2002). Human and experimental studies indicate that the fetus and infant are more sensitive than adults to diverse environmental toxicants, including lead, mercury, environmental tobacco smoke (ETS), polycyclic aromatic hydrocarbons (PAHs), and pesticides (National Research Council 1993; Neri et al. 2006; Perera et al. 2005b; Whyatt and Perera 1995; World Health Organization 1986). Urban minority populations represent high-risk groups for adverse health and developmental outcomes (Claudio et al. 1999; Federico and Liu 2003; New York City Department of Health 1998; Perera et al. 2002). Although urban air pollution crosses geographic and socioeconomic boundaries, these same populations are likely to be more heavily exposed to indoor and outdoor air pollution and pesticides (Breysse et al. 2005; Olden and Poje 1995; Perera et al. 2002). As reported previously, the present study cohort has had substantial although variable exposure to multiple contaminants during pregnancy, with 100% of subjects having exposure to PAHs and pesticides in the air during pregnancy and 40% reporting ETS exposure (Perera et al. 2003; Rauh et al. 2004; Whyatt et al. 2002). PAH exposure in this urban cohort of nonsmokers is largely due to traffic sources and ETS (which was controlled for in analyses).

To our knowledge, there have been no prior human studies of the effect of prenatal exposure to airborne PAHs on child development. However, prenatal exposure to ETS has been associated with reduced fetal growth and cognitive functioning (Martinez et al. 1994; Rauh et al. 2004; Schuster and Ludwig 1994; Sexton et al. 1990; Windham et al. 1999; Yolton et al. 2005). Associations have been observed between prenatal exposure to the pesticide chlorpyrifos (CPF) and neurodevelopmental outcomes in experimental systems (Aldridge et al. 2005). Lead and mercury are known developmental toxicants affecting fetal development (Agency for Toxic Substances and Disease Registry 1999; Canfield et al. 2003; Grandjean et al. 1997; Lanphear et al. 2000).

In addition to being genotoxic and carcinogenic, PAHs such as benzo[*a*]pyrene (BaP) are endocrine disruptors (Bostrom et al. 2002; Bui et al. 1986; Kazeto et al. 2004). Prior laboratory and human studies in Central Europe and in our New York City cohort indicate that transplacental exposure to PAHs is associated with adverse birth outcomes (Barbieri et al. 1986; Bui et al. 1986; Dejmek et al. 2000; Legraverend et al. 1984; Perera et al. 1998, 2005a). In the present analysis, we evaluated the effects of prenatal exposure to airborne PAHs, estimated by personal air sampling of the mother during pregnancy, on mental and psychomotor development of children through 36 months of age, controlling for physical, biologic, and psychosocial determinants of these outcomes.

## Materials and Methods

### Study subjects

The present cohort study is being conducted by the Columbia Center for Children’s Environmental Health (CCCEH) (Perera et al. 2003). The study was approved by the Institutional Review Board (IRB) of Columbia University. Dominican and African-American women (ethnicity classified by self-report) residing in Washington Heights, Central Harlem, and the South Bronx, New York, who registered at the obstetrics/gynecology clinics at New York Presbyterian Medical Center and Harlem Hospital by the 20th week of pregnancy were approached in the clinics for consent. At that time, the women agreeing to participate in the prospective cohort study signed the IRB-approved consent form. Eligible women were nonsmokers during the current pregnancy; were free of diabetes, hypertension, and known HIV; had no documented or reported drug abuse; and had resided in the area for at least 1 year. At the time of this report, of 648 consenting and eligible mother–infant pairs, 536 were still participating in the cohort study; 271 children had reached 3 years of age. The retention rate for the full cohort was 83% at the 3-year follow-up. There were no significant differences between women retained in the study versus those who were lost to follow-up, on maternal age, ethnicity, marital status, education, income, gestational age, or birth weight of the newborn.

In this report we focus on the 183 children 3 years of age who had valid prenatal PAH monitoring data, all three annual developmental assessments, prenatal questionnaire data on ETS, measurements of cotinine in maternal and cord blood samples ≥25 ng/mL (to exclude the possibility that the mother was an active smoker), and CPF level in cord blood. This group did not differ in any of the maternal or infant characteristics or prenatal exposures in Table 1 from the 80 children 3 years of age excluded from the analysis because of missing data. Of these, 64 children were excluded because of missing developmental testing data.

### Personal interview

A 45-min questionnaire was administered by a trained bilingual interviewer during the last trimester of pregnancy (Perera et al. 2003). The questionnaire elicited demographic information, residential, health, and environmental history, including active and passive smoking [household members who smoke and estimated cigarettes smoked per day by smoker(s)], and socioeconomic information related to income and education. Postnatal interviews were administered at 6 months, annually, and every 3–6 months in between to determine any changes in residence, exposure to ETS, and other health or environmental conditions.

### Prenatal personal PAH assessment

During the third trimester of pregnancy, personal monitoring was carried out as previously described (Perera et al. 2003). Vapors and particles ≥2.5 μg in diameter were collected on a precleaned quartz microfiber filter and a pre-cleaned polyurethane foam cartridge backup. The samples were analyzed at Southwest Research Institute (San Antonio, TX) for benz[*a*]anthracene, chrysene, benzo[*b*]fluroanthene, benzo[*k*]fluroanthene, BaP, indeno-[1,2,3-*cd*]pyrene, disbenz[*a,h*]anthracene, and benzo[*g,h,i*]perylene as described by Tonne et al. (2004). For quality control, each personal monitoring result was assessed as to accuracy in flow rate, time, and completeness of documentation. All of the 183 subjects had samples of acceptable quality.

### Biologic sample collection and analysis

A sample of umbilical cord blood (30–60 mL) was collected at delivery by syringing blood into a heparinized syringe to avoid clotting. A sample of maternal blood (30–35 mL) was collected within 2 days postpartum into heparinized Vacutainer tubes (BD Medical, Franklin Lakes, NJ) by hospital staff. Samples were processed at the CCCEH laboratory, and portions were sent to the Environmental Health Laboratory at the Centers for Disease Control and Prevention (CDC; Atlanta, GA) for analysis of cotinine, heavy metals, and pesticides. Plasma cotinine was analyzed using high-performance liquid chromatography atmospheric-pressure ionization tandem mass spectrometry as described by Bernert et al. (1997, 2000). Plasma levels of CPF were analyzed using isotope-dilution gas chromatography–high-resolution mass spectrometry as described by Barr et al. (2002). In a subset (*n* = 135) of subjects, lead was analyzed by inductively coupled plasma mass spectrometry (CDC 2003).

### Information on pregnancy outcomes

Information was abstracted by the research workers from mothers’ and infants’ medical records after delivery, including gestational age at birth, infant sex, birth weight, length, head circumference, infant malformations, and pregnancy complications. Gestational age was based on medical records for almost all subjects. Where those data were missing, gestational age was calculated from the last menstrual period.

### Measures of child behavior and neurodevelopment

We used the Bayley Scales of Infant Development–Revised (BSID-II) to assess cognitive and psychomotor development at 12, 24, and 36 months of age (Bayley 1993). The BSID-II is the most widely used norm-referenced developmental test for young children, can be used to diagnose developmental delay, and is known to be sensitive to the developmental effects of toxic exposures such as low-level intrauterine lead. The stability of cognitive assessments during the first few years of life is limited, but the predictive power increases after 2 years. When administered at 3 years of age, the BSID-II has moderate predictive power for subsequent intelligence and school performance and is clinically useful for the identification of children performing in the subnormal range (Bayley 1993; Burchinal et al. 2000; Sternberg et al. 2001). Each test yields a developmental quotient (raw score/chronologic age), which generates a mental development index (MDI) and a corresponding psychomotor development index (PDI). In addition, children are classified as normal (> 85), moderately delayed (> 70 and ≥85), or severely delayed (≥70) based on standardized cut-points. Each child was tested under controlled conditions at the CCCEH by a bilingual research assistant, trained and checked for reliability. In the present study, the interrater reliability for the 24-month MDI was *r* = 0.92, based on double scoring of a random 5% of the sample (Rauh et al. 2004). One hundred eighty-one children had complete MDI at 1, 2, and 3 years of age; 181 had complete data on PDI, and 183 had either complete MDI or PDI.

Behavior problems were measured by maternal report on the 99-item Child Behavior Checklist (CBCL) for children 1.5–5 years of age, which collects information on child behaviors occurring in the past 2 months (Achenbach and Rescorla 2000). The CBCL is well validated, easy to administer, and useful as a screen for behavior problems. The Total Problems (T) score is the sum of the scores on the specific problem items plus the highest score on any additional problems entered by the respondent for the open-ended item 100, and is computed by summing the scores for the problems. T scores > 63 (> 90th percentile) represent the clinical range, and T scores between 60 and 63 (83rd to 90th percentile) represent the borderline range. The CBCL also yields scales derived from the *Diagnostic and Statistical Manual of Mental Disorders* 2000 that are intended to approximate clinical diagnoses, including affective, anxiety, pervasive developmental, attention deficit/hyperactivity, and oppositional defiant problems. All sub-scales are scored continuously and also categorically using a borderline or clinical cut-point corresponding to the 98th percentile for each domain. One hundred sixty-eight children of the children with Bayley scores also had CBCL data.

Maternal nonverbal intelligence was measured by the Test of Non-Verbal Intelligence, second edition (Brown et al. 1990), a 15-min, language-free measure of general intelligence, relatively stable and free of cultural bias. The test was administered when the child was 3 years of age. The quality of the proximal caretaking environment was measured by the Home Observation for Measurement of the Environment (Caldwell and Bradley 1979), administered at 3 years of age. The instrument assesses physical and interactive home characteristics (Bradley et al. 1989), is predictive of developmental scores in early childhood, and has been widely used in studies of neurotoxicity (e.g., Bellinger et al. 1988).

### Statistical analysis

As in prior analysis (Perera et al. 2003), a composite PAH variable was computed from the eight inter-correlated PAH air concentration measures (*r* values ranging from 0.34 to 0.94; all *p*-values < 0.001 by Spearman’s rank). This variable was dichotomized at the fourth quartile (4.16 ng/m^3^) to obtain a measure of high/low exposure that is more robust than the continuous variable. The CPF variable was also dichotomized at the fourth quartile as previously described (Whyatt et al. 2004). The concentration of lead in cord blood was treated as a continuous variable.

We estimated the associations between pre-natal PAH exposure (high/low) and developmental scores (cognitive and psychomotor) for 12, 24, and 36 months of age using multiple linear regression for continuous outcomes (MDI and PDI) and logistic regression for categorical outcomes (likelihood of being classified as developmentally delayed). We estimated associations between prenatal PAH exposure (high/low) and behavior problems in the clinical range at 36 months of age using logistic regression. We used general estimated equation (GEE) (Liang and Zeger 1986) to estimate the size of the PAH effect over time (through 36 months) and at specific time points. To evaluate trends over time, the model compares the two exposure groups in terms of the difference in MDI scores obtained at 1 year of age (baseline) versus 2 years and 1 year versus 3 years, respectively. GEE has the advantage of requiring fewer assumptions than other methods; thus, the results of GEE are more robust. All effect estimates, 95% confidence intervals (CIs), and *p*-values (α = 0.05) were generated using SPSS (version 11.5; SPSS Inc., Chicago, IL) and SAS (version 9.0; SAS Institute Inc., Cary, NC). Covariates were retained in the models as potential confounders if they exhibited a relationship (*p* ≥0.1) with motor or mental development, regardless of their association with PAH exposure. The final models included an indicator for PAH exposure, the child’s exact age at test administration, child’s sex, ethnicity, gestational age at birth, quality of the home (caretaking) environment, and prenatal exposure to ETS and CPF measured as described above. In addition, the possible confounding of prenatal exposure by lead was tested in the subset of 135 children with available data. Interactions of PAH exposure with other independent variables were tested as appropriate.

We assessed potential mediation of the association between PAH exposure and development over time by including those fetal growth parameters previously shown to be affected by prenatal PAH exposure (birth weight and head circumference) in the models. If the estimate of the PAH effect on neurodevelopment was attenuated in the presence of a fetal growth parameter, mediation was considered to be present.

## Results

Table 1 describes the characteristics of the sample stratified by level of PAH exposure. There were no significant differences between high- and low-exposure groups except for the home environment, which was less favorable in the high-exposure group. Prenatal PAH exposures averaged 3.49 ng/m^3^, with a range of 0.65–36.47 ng/m^3^; 39.3% of children had prenatal ETS exposure. Table 2 shows the mean ± SD or proportion for the developmental outcomes. These include the indices of performance on the BSID-II, including MDI, PDI, proportion moderately delayed on the mental index, and proportion moderately delayed on the motor index for each of the exposure groups at 12, 24, and 36 months of age. Table 2 also shows the mean ± SD for the CBCL score for total behavior problems.

In univariate analysis of children with all three developmental measures, prenatal exposure to PAHs was significantly associated with MDI at age 3 (β= –4.68; 95% CI, –8.13 to –1.24; *p* = 0.01; *n* = 263) but not MDI at 1 or 2 years of age nor with PDI. Table 3 shows the age-specific parameter estimates for prenatal PAH exposure effects on mental and motor development, by multiple regression adjusting for the covariates as described in statistical analysis (*n* = 181). There was a significant effect of prenatal PAH exposure on MDI at age 3 (β= –5.69; 95% CI, –9.05 to –2.33; *p* < 0.01) but not 1 or 2 years of age. PDI was not associated with PAH exposure at any age. Altogether, the exposures and covariates accounted for approximately 31.2% of the variance in MDI scores at 36 months. None of the interaction terms of PAHs and the sociodemographic or exposure variables was significant. There was no significant interaction between PAH exposure level and home environment, suggesting that the magnitude of the 36-month prenatal PAH effect is not affected by the quality of the caretaking environment.

In univariate logistic regression analysis, the likelihood of a child experiencing moderate mental developmental delay at 3 years of age was significantly increased as a function of prenatal PAH exposure (odds ratio = 2.05; 95% CI, 1.15–3.63; *p* = 0.01), but again, the relationship was not seen at 1 or 2 years of age nor with psychomotor developmental delay. Table 4 shows the results of the logistic regression analysis adjusting for the relevant covariates as described in statistical analyses. The odds ratio for delayed mental development at 36 months of age was 2.89 (95% CI, 1.33–6.25; *n* = 181). PAH exposure was not a significant predictor of psychomotor development. There were no significant interactions between PAHs and the other covariates in logistic regression.

**Figure 1:**
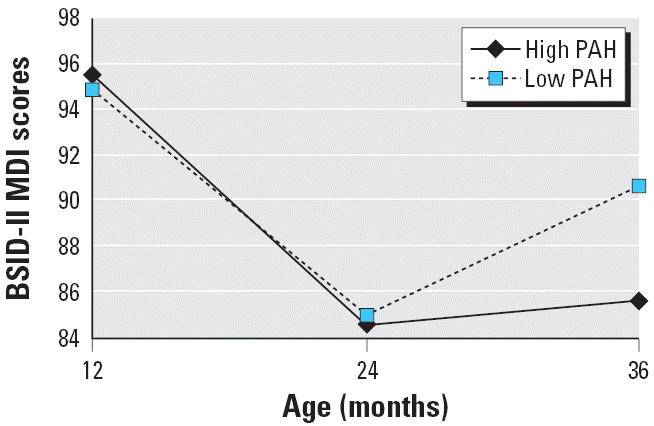
Estimated effects of prenatal PAH exposure on cognitive development in children 12 months through 36 months of age by GEE. The model was adjusted for the child’s exact age at test administration, child’s sex, ethnicity, gestational age at birth, quality of the (caretaking) home environment, and prenatal exposure to ETS and CPF.

Table 5 and Figure 1 show the results of the GEE analysis of PAH effects on cognitive development over the 3-year follow-up period. The significant age × PAH effect on mental development (*p* = 0.01) confirms the age-specific regression findings showing that an adverse impact of prenatal PAH exposure on this developmental domain was seen only over time. For motor development, there was no significant relationship (Table 5). At 3 years of age, the decrease in MDI from baseline was significantly greater for high-exposed compared to low-exposed children (*p* = 0.01), whereas the difference was not significant at 2 years of age. The results of analyses using the continuous measure of PAHs were generally similar but less significant. Inclusion of fetal growth parameters (birth weight or head circumference) did not alter the effect of PAHs on 36 month MDI. Inclusion of cord lead as a covariate did not materially alter the association between PAHs and development. The results of regression of total CBCL behavior problems on prenatal PAH exposure were not significant, nor were any of the subscales significantly related to PAH exposure.

## Discussion

Previous results from this cohort have indicated that exposure to PAH air pollutants during pregnancy has produced DNA damage and impaired fetal growth (Perera et al. 2003, 2005a, 2005b). The present analysis suggests a further impact of prenatal PAH exposure on cognitive development. The infants who had been exposed prenatally to the highest PAH levels scored significantly lower on MDI at 3 years of age than did those with lower levels of PAH exposure. Although the adjusted mean MDI scores of the high- and low-exposed PAH groups differed by only 5.69 points, among the highly exposed children the odds of having MDI scores < 85 at 3 years of age (indicating moderate delay) were 2.89 times greater than the odds among unexposed children. This suggests that more exposed children are potentially at risk for performance deficits (language, reading, and math) in the early school years. In fact, developmentally delayed children are eligible for early intervention services designed for children who are at possible risk for early school failure. The observed magnitude of the PAH effect on early development in this study is comparable to that reported for low-level lead exposure (Schwartz 1994). In this study, there was no effect of PAHs on cognitive development at 1 and 2 years of age, nor were psychomotor development and behavioral problems associated with PAHs. The impact of PAHs on mental development at 3 years of age does not appear to be mediated by birth weight or head circumference, fetal growth parameters previously shown to be associated with prenatal PAH exposure in this cohort (Perera et al. 2003). The children are being followed to 7–8 years of age, so subsequent testing will provide a picture of the developmental trajectory of this group.

To our knowledge, there have been no prior studies of the role of prenatal exposure to airborne PAHs in child neurodevelopment. However, a study in the Czech Republic reported that schoolchildren in the district of Teplice, which had higher levels of PAHs and other air pollutants from coal burning than did the district of Prachatrice, had a significantly higher teacher referral rate for clinical assessment compared to Prachatrice, although most objective performance measures did not differ (Šrám et al. 1996).

The mechanisms by which PAHs might affect the developing brain are not known. However, fetal toxicity may be caused by antiestrogenic effects (Bui et al. 1986) binding to the human aryl hydrocarbon receptor to induce P450 enzymes (Manchester et al. 1987), DNA damage resulting in activation of apoptotic pathways (Meyn 1995; Nicol et al. 1995; Wood and Youle 1995), or binding to receptors for placental growth factors resulting in decreased exchange of oxygen and nutrients (Dejmek et al. 2000).

In the present cohort, the mean developmental scores are below average for the normed population, reflecting the low-income nature of the catchment area in this study (e.g., Bradley and Corwyn 2002; Burchinal et al. 1997; Luster and McAdoo 1996). In addition, children from homes with low levels of stimulation and mother–child interaction had significantly lower scores on the Bayley cognitive scales, independent of PAH exposure.

This study has the advantage of being based on individual prenatal exposure data from personal monitoring and biomarker analyses, as well as extensive medical record and questionnaire data. However, it is limited by the modest sample of subjects for whom data from all relevant domains are currently available. Moreover, relationships observed in low-income minority women might be different in women of other races or ethnic, cultural, or socioeconomic backgrounds. Further, it is possible that high levels of PAHs may be associated with living near an exposure source such as a bus route or garage leading to some uncontrolled confounding by socioeconomic status even within our low-income population.

Another limitation is that we lacked air monitoring data for all three trimesters and were therefore not able to compare exposures across these three periods. We also lacked post-natal personal air monitoring data for PAHs and were unable to control directly for postnatal PAH exposure. However, when we controlled for change in residence as a proxy for variation in PAH exposure between the pre-and postnatal periods, the effects of prenatal PAHs remained. We also lacked postnatal environmental lead exposure data, which may have been a critical period of exposure to this known developmental toxicant. Additional studies are needed to tease apart the effects of prenatal and postnatal exposure to PAHs and to confirm the present findings.

## Conclusion

This study provides evidence that environmental PAHs at levels recently encountered in the air of New York City may adversely affect cognitive development of children. The results require confirmation but are of potential concern because compromised mental performance in the preschool years is an important precursor of subsequent educational performance deficits. PAHs are widespread in urban environments worldwide largely as a result of fossil fuel combustion. Fortunately, airborne PAH concentrations can be reduced through currently available pollution controls, greater energy efficiency, and the use of alternative energy sources (Wong et al. 2004).

## Figures and Tables

**Table 1:** Characteristics of the study population by PAH exposure level (*n* = 183).^a^

	Prenatal PAH exposure level	
Characteristic	High exposure^b^ (*n* = 42)	Low exposure^b^ (*n* = 141)
Maternal characteristics		
Ethnicity (%)		
African American	40.5	47.5
Latino	59.5	52.5
Age (years)	25.03 ± 4.79	24.80 ± 5.53
Married (%)	11.9	15.1
No high school degree (%)	47.6	31.9
Maternal intelligence quotient	84.37 ± 10.27	86.27 ± 13.45
Caretaking home environment	37.55 ± 5.58*	39.82 ± 5.82*
Infant characteristics
Birth weight (g)	3,357.44 ± 529.69	3,413.16 ± 462.47
Birth length (cm)	50.73 ± 2.72	50.79 ± 3.85
Birth head circumference (cm)	34.01 ± 1.69	34.34 ± 1.95
Gestational age (week)	39.17 ± 1.25	39.40 ± 1.38
Percent male	47.6	45.4
Prenatal exposure		
ETS [at least one smoker in house (%)]	38.1	39.7
CPF (pg/g)	5.94 ± 11.44	3.56 ± 4.45
Cord lead (μg/dL)^c^	1.01 ± 0.69	1.08 ± 0.79

Values are mean ± SD or percent.

a Includes subjects with MDI and/or PDI (*n* = 183).

b High exposure was defined as the fourth quartile of PAH; low exposure was defined as all others (quartiles 1, 2, 3). There was no difference between the two exposure groups with respect to any of the characteristics, except for the home environment.

c Cord lead was available in a subset of 135.

* *p* < 0.05, by Wilcoxon rank-sum test.

**Table 2:** Mean ± SD and proportion for developmental and behavioral outcomes at 12, 24, and 36 months.

	Age at assessment		
Domain	12 months	24 months	36 months
MDI^a^	94.25 ± 9.45	85.01 ± 12.59	89.66 ± 11.21
PDI^b^	95.85 ± 12.17	97.40 ± 11.59	100.80 ± 13.21
Total behavior problems^c^	NA	NA	50.20 ± 10.47
Moderate mental development delay (%)^a^,^d^	14.9	48.1	33.1
Severe mental development delay (%)^a^,^e^	0.6	9.4	2.8
Moderate psychomotor development delay (%)^b^,^f^	14.4	13.3	10.5
Severe psychomotor development delay (%)^b^,^g^	1.7	1.7	1.7

NA, not applicable.

a *n* = 181 with all 3 years of MDI data.

b *n* = 181 with all 3 years of PDI data.

c *n* = 168 with all 3 years of MDI and/or PDI and behavioral data (T score).

d MDI < 85.

e MDI < 70.

f PDI < 85.

g PDI < 70.

**Table 3:** Multiple linear regression models testing effects of prenatal PAH exposure at 12, 24, and 36 months using MDI and PDI^a^ (*n* = 181).

	Model 1: 12 months		Model 2: 24 months		Model 3: 36 months	
	β	*p*-Value	β	*p*-Value	β	*p*-Value
MDI						
Constant	74.52	< 0.01	37.73	0.16	52.79	0.02
PAHs	0.48	0.78	–1.73	0.41	–5.69	< 0.01
Ethnicity (1 = African American; 0 = others)	0.24	0.88	6.66	< 0.01	6.34	< 0.01
Sex (1 = male)	–2.09	0.14	–4.51	0.01	–2.20	0.13
Gestational age	0.38	0.49	0.95	0.16	0.41	0.44
Home environment	0.13	0.30	0.28	0.08	0.54	< 0.01
PDI						
Constant	95.34	< 0.01	109.77	< 0.01	54.00	0.06
PAHs	1.32	0.55	–2.08	0.32	–0.97	0.68
Ethnicity (1 = African American; 0 = others)	–2.79	0.16	1.87	0.32	4.45	0.03
Sex (1 = male)	1.12	0.54	0.52	0.76	–1.24	0.52
Gestational age	0.15	0.82	–0.52	0.43	0.95	0.19
Home environment	–0.10	0.56	0.16	0.30	0.25	0.15

a Models were also adjusted for prenatal ETS and CPF. Further inclusion of maternal IQ and maternal education as covariates did not alter the results.

**Table 4:** Logistic regression models testing effects of prenatal PAH exposure on the odds of mental and psychomotor development delay at 12, 24, and 36 months^a^ (*n* = 181).

	Model 1: 12 months			Model 2: 24 months			Model 3: 36 months		
	β	*p*-Value	Exp(β)	β	*p*-Value	Exp(β)	β	*p*-Value	Exp(β)
Dependent variable: moderate delay (MDI < 85)									
Constant	2.65	0.67	14.12	3.04	0.53	20.92	6.68	0.24	798.33
PAHs	−0.19	0.71	0.82	−0.16	0.68	0.86	1.06	0.01	2.89
Ethnicity (1 = African American; 0 = others)	0.32	0.50	1.37	−0.90	0.01	0.41	−0.77	0.06	0.46
Sex (1 = male)	0.66	0.12	1.94	0.84	0.01	2.31	0.50	0.16	1.65
Gestational age	−0.07	0.67	0.93	−0.02	0.89	0.98	−0.10	0.47	0.90
Home environment	−0.05	0.19	0.95	−0.06	0.03	0.94	−0.10	< 0.01	0.90
Dependent variable: moderate delay (PDI < 85)									
Constant	−1.50	0.82	0.22	−1.11	0.87	0.33	−2.30	0.79	0.10
PAHs	−0.92	0.16	0.40	0.41	0.41	1.51	−0.22	0.72	0.80
Ethnicity (1 = African American; 0 = others)	0.39	0.41	1.48	−0.21	0.67	0.81	−0.65	0.28	0.52
Sex (1 = male)	−0.18	0.68	0.83	−0.42	0.35	0.66	0.01	0.98	1.01
Gestational age	−0.01	0.97	0.99	−0.02	0.89	0.98	0.04	0.85	1.04
Home environment	−0.01	0.88	0.99	0.01	0.86	1.01	−0.05	0.25	0.95

a Models were also adjusted for prenatal ETS and CPF. Further inclusion of maternal IQ and maternal education as covariates did not alter the results.

**Table 5:** Cognitive mental development in children 12 months through 36 months of age by GEE (*n* = 543 measurements).^a^

	MDI		PDI	
	β	*p*-Value	β	*p*-Value
Intercept	60.46	< 0.01	84.76	< 0.01
24 months	−9.87	< 0.01	−2.12	0.20
36 months	−4.29	0.01	3.53	0.08
PAHs	0.69	0.66	1.89	0.35
24 months × PAHs	−1.12	0.62	−3.14	0.16
36 months × PAHs	−5.57	0.01	−4.65	0.08
Ethnicity (1 = African American; 0 = others)	−0.23	0.88	−3.38	0.07
24 months × ethnicity	6.80	< 0.01	5.71	0.01
36 months × ethnicity	7.14	< 0.01	7.91	< 0.01
Sex (1 = male)	−2.80	0.01	0.08	0.95
Gestational age	0.56	0.16	0.23	0.62
Home environment	0.31	< 0.01	0.11	0.39

a Models were also adjusted for prenatal ETS and CPF.
